# Crystal structures, DFT studies and UV–visible absorption spectra of two anthracenyl chalcone derivatives

**DOI:** 10.1107/S2056989018013087

**Published:** 2018-09-28

**Authors:** Dian Alwani Zainuri, Ibrahim Abdul Razak, Suhana Arshad

**Affiliations:** a X-ray Crystallography Unit, School of Physics, Universiti Sains Malaysia, 11800 USM, Penang, Malaysia

**Keywords:** crystal structure, anthracene, DFT, UV–Vis

## Abstract

The crystal structures of (*E*)-1-(anthracen-9-yl)-3-(3*H*-indol-2-yl)prop-2-en-1-one, C_25_H_17_NO, and (*E*)-1-(anthracen-9-yl)-3-[4-(di­methyl­amino)­naphthalen-1-yl]prop-2-en-1-one, C_29_H_23_NO, are reported. In each case the anthracene ring system and pendant ring system are almost perpendicular to each other [dihedral angles = 75.57 (7)° and 70.26 (10)°, respectively]. In the extended structures, weak N—H⋯O, C—H⋯O and C—H⋯π inter­actions influence the centrosymmetric crystal packing. Density functional theory calculations were carried out using a 6–311 G++(d,p) basis set and the calculated structures are in good agreement with the crystal structures. The compounds were also characterized by UV–Vis absorption spectroscopy and the smallest (HOMO–LUMO) energy gaps of 2.89 and 2.54 eV indicate the enhanced non-linear responses (inter­molecular charge transfers) of these systems.

## Abstract 2

In each structure, the anthracene ring system and pendant ring system are almost perpendicular to each other [dihedral angles = 75.57 (7) and 70.26 (10)°]. In the extended structures, weak N—H⋯O, C—H⋯O and C—H⋯π inter­actions influence the centrosymmetric crystal packing.

## Chemical context   

Organic mol­ecules are used extensively in many NLO applications such as electro-optic modulation, THz wave generation and optical power limiting (He *et al.*, 2008 ▸). These properties originate from their inherent large mol­ecular hyperpolarizabilities arising from delocalized π-electrons along the length of the mol­ecule. Various design strategies have been established to make new organic mol­ecules with larger polarizabilities such as asymmetric *D*–π–*A*, symmetric *D*–π–*D*, *A*–π–*A etc* (*D* = donor, *A* = acceptor). π-Conjugated mol­ecular materials with fused rings are the focus of considerable inter­est in the emerging area of organic electronics, since the combination of good charge-carrier mobility and high stability might lead to potential optoelectronic applications (Wu *et al.*, 2010 ▸). A chalcone mol­ecule with a π-conjugated system provides a large charge-transfer axis with appropriate substituent groups on the two terminal aromatic rings (D’Silva *et al.*, 2011 ▸).
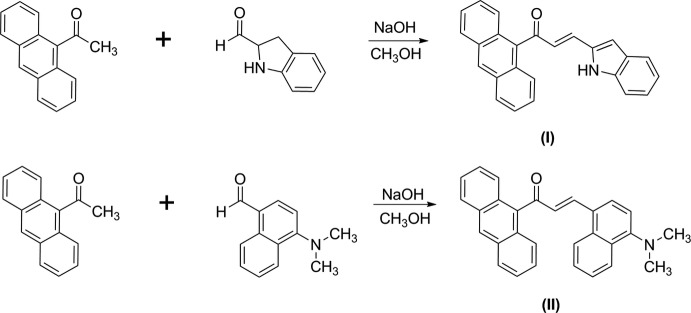


Previously, we have reported several anthracenyl chalcone derivatives with various substituent groups (Zainuri *et al.*, 2018*a* ▸,*b* ▸,*c* ▸,*d* ▸) and as part of our ongoing studies of such systems, we now describe the syntheses, crystal structures, UV–visible spectroscopy and theoretical calculations on a combination of an anthracene fused-ring system (strong electron donor) and the substituents indoline (I) and *N*,*N*-di­methyl­naphthalen-1-amine (II), which act as a strong electron donor at the terminal ring derivatives, establishing a *D*–π–*D* system.

## Structural commentary   

**Figure 1:**
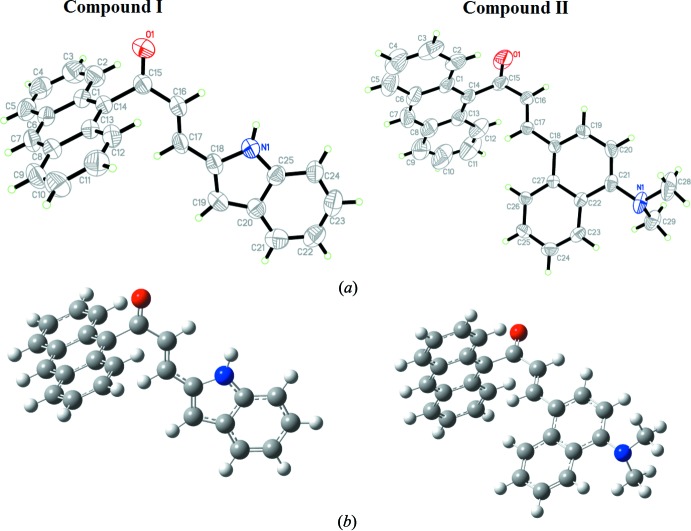
(*a*) The mol­ecular structure of compounds (I) and (II) and (*b*) the structures optimized at the DFT/B3LYP 6–311++G(d,p) level of theory.

The mol­ecular structures of (I) and (II) are shown in Fig. 1 ▸ *a*: both crystallize in centrosymmetric space groups [*P* 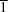 for (I) and *P*2_1_/*c* for (II)]. Each compound is made up of an anthracene ring system with the substituents indoline and *N*,*N*-di­methyl­naphthalen-1-amine for (I) and (II), respectively. The geometry-optimized structures are shown in Fig. 1 ▸ *b*. Selected calculated (Frisch *et al.*, 2009 ▸) structure parameters such as bond lengths, bond angles and torsion angles are listed in Table S1 in the supporting information from which it can be seen that the calculated parameters are in good agreement with the results obtained from the single-crystal refinements.

The enone moiety (O1/C15–C17) in (I) and (II) adopts an *s*-*trans* configuration with respect to the C15=O1 and C16=C17 bonds (Table S1). Both compounds (I) and (II) are twisted at the C14—C15 bond with C1—C14—C15—C16 torsion angles of −109.5 (2)° (experimental), −91.1° (DFT) and 96.4 (3)° (experimental), 96.0° (DFT), respectively. The bulkiness of the anthracene ring system gives rise to a highly twisted structure for both compounds (Zainuri *et al.*, 2018*a* ▸,*b* ▸,*c* ▸,*d* ▸). The atoms about the C17—C18 bonds are found to be nearly planar in (I) with the experimental and theoretical C16—C17—C18—C19 torsion angles being 180.0 (2) and 180.0°, respectively. In (II), the corresponding experimental and theoretical torsion angles are 17.4 (3) and 18.7°, respectively, showing that the mol­ecule is slightly twisted at the C17—C18 bond. It appears that the torsion-angle differences observed in (I) and (II) are due to the effect of the substituent group: in (I), the N—H grouping forms an inter­molecular N—H⋯O hydrogen bond, which locks the enone moiety and indoline ring into a near planar conformation.

Additionally, the enone moiety for (I) [O1/C15–C17, maximum deviation of 0.031 (18) Å at O1] forms dihedral angles of 72.9 (3) and 2.9 (3)° with the anthracene ring system [C1-C14, maximum deviation of 0.034 (3) Å at C5] and indoline moiety [N1/C18–C25, maximum deviation of 0.004 (3) Å at C25], respectively. In (II), the enone moiety [O1/C15–C17, maximum deviation of 0.067 (3) Å at O1] forms dihedral angles of 81.3 (3) and 18.7 (3)° with the anthracene ring system [C1–C14, maximum deviation of 0.035 (6) Å at C5] and naphthalene ring system [C18–C27, maximum deviation of 0.061 (3) Å at C19], respectively. Furthermore, the dihedral angles between the anthracene ring system and the indoline ring in (I) and naphthalene ring system in (II) are 75.57 (7) and 70.26 (10)°, respectively. The large dihedral angle may indicate the diminishing electronic effect between the anthracene groups through the enone bridge (Jung *et al.*, 2008 ▸).

## Supra­molecular features   

**Figure 2:**
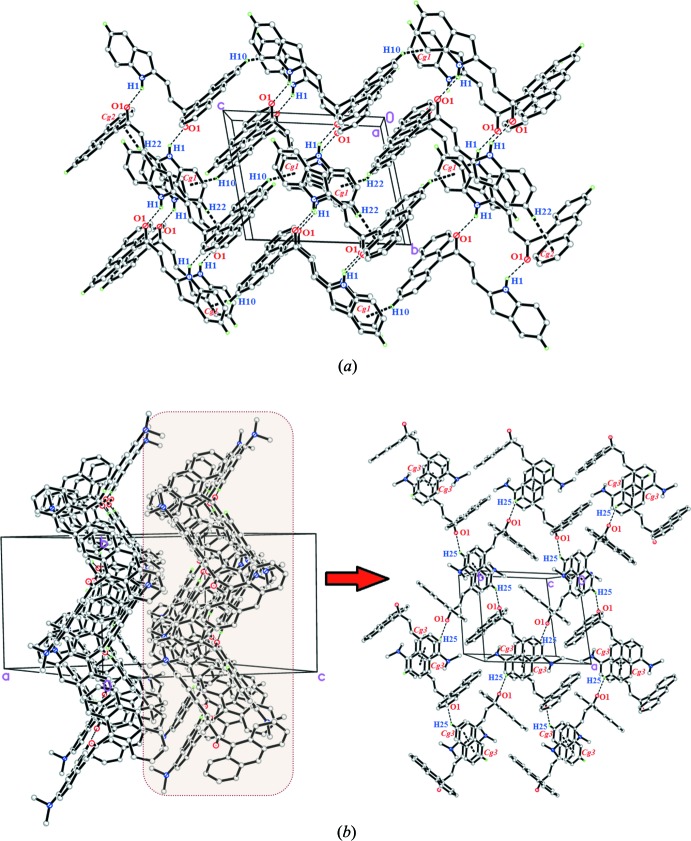
Packing diagram showing (*a*) weak N—H^⋯^O and C—H^⋯^π inter­actions in (I) and (*b*) weak C—H^⋯^O and π–π inter­actions in (II).

The crystal packing of (I) show that the mol­ecules are connected into centrosymmetric dimers *via* pairwise N—H⋯O hydrogen bonds (Table 1 ▸), forming *R^2^_2_*(14) loops (Fig. 2 ▸ *a*). These dimers are further linked into infinite sheets stacked along the *bc* plane. The weak C10—H10⋯*Cg*1 and C22—H22⋯*Cg*2 inter­actions also help to establish the packing. Overall, these links generate a three-dimensional supra­molecular network.

In (II), weak C25—H25⋯O1 bonds (Table 2 ▸) connect the mol­ecules into chains propagating along the *a*-axis direction (Fig. 2 ▸ *b*). A weak π–π inter­action (symmetry operation: −*x*, 1 − *y*, 1 − *z*) with a centroid–centroid distance of 3.9432 (16) Å between C22–C27 rings is also observed. Together these inter­actions generate a two-dimensional supra­molecular network propagating in the *ab* plane.

## Frontier mol­ecular orbital (FMO) and UV–vis absorption analysis   

For background to FMO analysis, which provides insight into electronic as well as optical properties of organic compounds, see: Ebenezar *et al.* (2013 ▸). In this study, the FMO analysis showed that the HOMO is mainly concentrated on the anthracene moiety for both compounds (see supporting information). Conversely, the LUMOs are mainly concentrated on the enone bridge and also their substituents [the indoline moiety in (I) and *N*,*N*-di­methyl­naphthalen-1-amine in (II)]. The HOMO–LUMO energy gap represents the lowest energy for inter­molecular charge transfer (ICT) where the *E*_HOMO_ and *E*_LUMO_ energies of the studied mol­ecules were calculated using the B3LYP/6-311G++(d,p) basis set. The calculated energy gaps (Fig. S1) are 3.16 eV in (I) and 3.19 eV in (II). These inter­molecular charge transfers result mainly from π–π* excitation.

**Figure 3:**
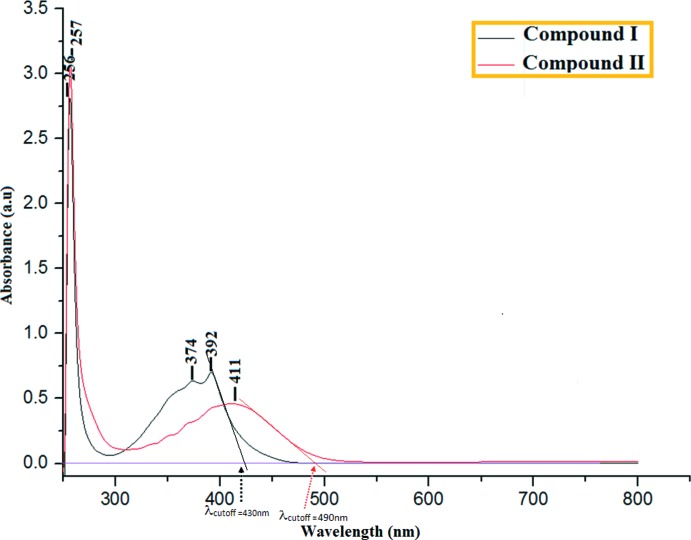
The UV–vis absorption spectra of compounds (I) and (II). For the extrapolation lines, see text.

The experimental UV–vis spectrum (Fig. 3 ▸) showed an absorption maximum at 392 nm (I) and 411 nm (II), which is in excellent agreement with the computed values of 396 nm (I) and 408 nm (II) in the gas phase. The observed absorption maxima of compound (I) and (II) can also be correlated with the HOMO–LUMO band gap. The experimental energy band gaps in (I) and (II) are estimated to be 2.89 eV and 2.54 eV, respectively, through a linear extrapolation of the low-energy side of the absorption maximum (see Fig. 3 ▸). These optical band-gap values indicate the potential suitability of this type of compound for optoelectronic applications (Tejkiran *et al.*, 2016 ▸). It may also be noted that these band gaps are comparable with inorganic materials used in optoelectronic device applications (Sathish *et al.*, 2015 ▸).

## Database survey   

A survey of Cambridge Structural Database (CSD, Version 5.39, last update Nov 2017; Groom *et al.*, 2016 ▸) revealed fused-ring substituted chalcones similar to the title compound. There are four compounds that have an anthracene-ketone substit­uent on the chalcone: 9-anthryl styryl ketone and 9,10-anthryl bis­(styryl ketone) were reported by Harlow *et al.* (1975 ▸), (2*E*)-1-(anthracen-9-yl)-3-[4-(propan-2-yl)phen­yl]prop-2-en-1-one was described by Girisha *et al.* (2016 ▸), while (*E*)-1-(anthracen-9-yl)-3-(2-chloro-6-fluoro­phen­yl)prop-2-en-1-one was reported by Abdullah *et al.* (2016 ▸). Zainuri *et al.* (2018 ▸) reported a structure with two anthrancene substituents on a chalcone, *viz*. (*E*)-1,3-bis­(anthracen-9-yl)prop-2-en-1-one. Others related compounds include 1-(anthracen-9-yl)-2-methyl­prop-2-en-1-one (Agrahari *et al.*, 2015 ▸) and 9-anthroylacetone (Cicogna *et al.*, 2004 ▸).

## Synthesis and crystallization   

9-Acetyl­anthrancene (0.5 mmol) was dissolved in methanol (20 ml) over about 10–15 min. Then, indoline-2-carbaldehyde (0.5 mmol) and 4-(di­methyl­amino)-1-naphthaldehyde (0.5 mmol) for compound (I) and (II), respectively, were added and the solutions were stirred for another 10–15 mins. Then, the solutions were dissolved in the presence of NaOH and stirred for another 4 h until the precipitates formed, at which point the reaction mixtures were poured into cold water (50 ml) and stirred for 10 min. The precipitated solids were filtered, dried and recrystallized from acetone solution to get the corresponding chalcones in the form of brown plates in each case.

## Refinement   

Crystal data collection and structure refinement details are summarized in Table 3 ▸. The hydrogen atom bounded to the nitro­gen [N—H=0.86 Å in (I)] and carbon [C—H = 0.93 Å in (I) and 0.93 and 0.96 Å in (II)] atoms were positioned geometrically and refined using a riding model with *U*_iso_(H) = 1.2 or 1.5*U*_eq_(C, N). A rotating group model was applied to the methyl groups.

## Supplementary Material

Crystal structure: contains datablock(s) I, II, global. DOI: 10.1107/S2056989018013087/hb7770sup1.cif

Structure factors: contains datablock(s) I. DOI: 10.1107/S2056989018013087/hb7770Isup2.hkl

Structure factors: contains datablock(s) II. DOI: 10.1107/S2056989018013087/hb7770IIsup3.hkl

Supporting information file. DOI: 10.1107/S2056989018013087/hb7770Isup4.cml

Supporting information file. DOI: 10.1107/S2056989018013087/hb7770IIsup5.cml

Supplementary figures and table. DOI: 10.1107/S2056989018013087/hb7770sup6.pdf

CCDC references: 1829208, 1824549

Additional supporting information:  crystallographic information; 3D view; checkCIF report

## Figures and Tables

**Table 1:** Hydrogen-bond geometry (Å, °) for (I) *Cg*1 and *Cg*2 are the centroids of the C20–C25 and C1–C6 rings, respectively.

*D*—H⋯*A*	*D*—H	H⋯*A*	*D*⋯*A*	*D*—H⋯*A*
N1—H1*A*⋯O1^i^	0.99 (3)	2.03 (3)	2.885 (3)	143 (3)
C10—H10*A*⋯*Cg*1^ii^	0.93	2.91	3.735 (3)	142
C22—H22*A*⋯*Cg*2^iii^	0.93	2.75	3.643 (3)	160

Symmetry codes: (i) 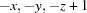; (ii) 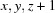; (iii) 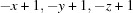.

**Table 2:** Hydrogen-bond geometry (Å, °) for (II)

*D*—H⋯*A*	*D*—H	H⋯*A*	*D*⋯*A*	*D*—H⋯*A*
C25—H25*A*⋯O1^i^	0.93	2.42	3.203 (3)	142

Symmetry code: (i) 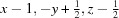.

**Table 3:** Experimental details

	(I)	(II)
Crystal data		
Chemical formula	C_25_H_17_NO	C_29_H_23_NO
*M*_r_	347.39	401.48
Crystal system, space group	Triclinic, *P* 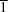	Monoclinic, *P*2_1_/*c*
Temperature (K)	296	296
*a*, *b*, *c* (Å)	8.542 (2), 9.500 (3), 11.521 (3)	12.6997 (7), 11.8029 (7), 18.3997 (9)
α, β, γ (°)	100.315 (6), 98.456 (6), 103.336 (6)	90, 127.901 (3), 90
*V* (Å^3^)	877.5 (4)	2176.3 (2)
*Z*	2	4
Radiation type	Mo *K*α	Mo *K*α
μ (mm^−1^)	0.08	0.07
Crystal size (mm)	0.33 × 0.14 × 0.07	0.62 × 0.54 × 0.23

Data collection		
Diffractometer	Bruker SMART APEXII Duo CCD	Bruker SMART APEXII Duo CCD
Absorption correction	Multi-scan (*SADABS*; Bruker, 2009 ▸)	Multi-scan (*SADABS*; Bruker, 2009 ▸)
*T*_min_, *T*_max_	0.891, 0.964	0.655, 0.946
No. of measured, independent and observed [*I* > 2σ(*I*)] reflections	23662, 4086, 1878	81111, 6334, 3215
*R*_int_	0.079	0.073
(sin θ/λ)_max_ (Å^−1^)	0.654	0.705

Refinement		
*R*[*F* ^2^ > 2σ(*F* ^2^)], *wR*(*F* ^2^), *S*	0.061, 0.177, 1.01	0.075, 0.222, 0.97
No. of reflections	4086	6334
No. of parameters	248	282
H-atom treatment	H atoms treated by a mixture of independent and constrained refinement	H-atom parameters constrained
Δρ_max_, Δρ_min_ (e Å^−3^)	0.19, −0.16	0.34, −0.22

Computer programs: *APEX2* and *SAINT* (Bruker, 2009 ▸), *SHELXL2014/7* (Sheldrick, 2015 ▸), *SHELXTL* (Sheldrick, 2008 ▸) and *PLATON* (Spek, 2009 ▸).

## supplementary crystallographic information

### (E)-1-(Anthracen-9-yl)-3-(3H-indol-2-yl)prop-2-en-1-one (I) Crystal data

**Table:**

C_25_H_17_NO	*Z* = 2
*M**_r_* = 347.39	*F*(000) = 364
Triclinic, *P*1	*D*_x_ = 1.315 Mg m^−^^3^
*a* = 8.542 (2) Å	Mo *K*α radiation, λ = 0.71073 Å
*b* = 9.500 (3) Å	Cell parameters from 1617 reflections
*c* = 11.521 (3) Å	θ = 2.5–19.3°
α = 100.315 (6)°	µ = 0.08 mm^−^^1^
β = 98.456 (6)°	*T* = 296 K
γ = 103.336 (6)°	Plate, brown
*V* = 877.5 (4) Å^3^	0.33 × 0.14 × 0.07 mm

### (E)-1-(Anthracen-9-yl)-3-(3H-indol-2-yl)prop-2-en-1-one (I) Data collection

**Table:**

Bruker SMART APEXII Duo CCD diffractometer	1878 reflections with *I* > 2σ(*I*)
Radiation source: fine-focus sealed tube	*R*_int_ = 0.079
φ and ω scans	θ_max_ = 27.7°, θ_min_ = 1.8°
Absorption correction: multi-scan (*SADABS*; Bruker, 2009)	*h* = −11→11
*T*_min_ = 0.891, *T*_max_ = 0.964	*k* = −12→12
23662 measured reflections	*l* = −15→15
4086 independent reflections	

### (E)-1-(Anthracen-9-yl)-3-(3H-indol-2-yl)prop-2-en-1-one (I) Refinement

**Table:**

Refinement on *F*^2^	Primary atom site location: structure-invariant direct methods
Least-squares matrix: full	Hydrogen site location: mixed
*R*[*F*^2^ > 2σ(*F*^2^)] = 0.061	H atoms treated by a mixture of independent and constrained refinement
*wR*(*F*^2^) = 0.177	*w* = 1/[σ^2^(*F*_o_^2^) + (0.0757*P*)^2^] where *P* = (*F*_o_^2^ + 2*F*_c_^2^)/3
*S* = 1.01	(Δ/σ)_max_ < 0.001
4086 reflections	Δρ_max_ = 0.19 e Å^−^^3^
248 parameters	Δρ_min_ = −0.16 e Å^−^^3^
0 restraints	

### (E)-1-(Anthracen-9-yl)-3-(3H-indol-2-yl)prop-2-en-1-one (I) Special details

**Table:**

Experimental. The following wavelength and cell were deduced by SADABS from the direction cosines etc. They are given here for emergency use only: CELL 0.71103 8.587 9.545 11.572 100.259 98.429 103.381
Geometry. All esds (except the esd in the dihedral angle between two l.s. planes) are estimated using the full covariance matrix. The cell esds are taken into account individually in the estimation of esds in distances, angles and torsion angles; correlations between esds in cell parameters are only used when they are defined by crystal symmetry. An approximate (isotropic) treatment of cell esds is used for estimating esds involving l.s. planes.

### (E)-1-(Anthracen-9-yl)-3-(3H-indol-2-yl)prop-2-en-1-one (I) Fractional atomic coordinates and isotropic or equivalent isotropic displacement parameters (Å^2^)

**Table:**

	*x*	*y*	*z*	*U*_iso_*/*U*_eq_	
N1	0.1118 (2)	0.3227 (2)	0.46075 (17)	0.0521 (5)	
H1A	0.029 (4)	0.225 (3)	0.440 (3)	0.120 (11)*	
O1	0.1474 (2)	−0.08316 (18)	0.69408 (16)	0.0727 (6)	
C1	0.5129 (3)	0.0493 (2)	0.8102 (2)	0.0464 (6)	
C2	0.5257 (3)	−0.0546 (3)	0.7111 (2)	0.0638 (7)	
H2A	0.4392	−0.0897	0.6452	0.077*	
C3	0.6616 (4)	−0.1046 (3)	0.7097 (3)	0.0748 (8)	
H3A	0.6675	−0.1724	0.6424	0.090*	
C4	0.7930 (3)	−0.0563 (3)	0.8075 (3)	0.0738 (8)	
H4A	0.8847	−0.0933	0.8060	0.089*	
C5	0.7867 (3)	0.0448 (3)	0.9049 (3)	0.0643 (7)	
H5A	0.8750	0.0775	0.9696	0.077*	
C6	0.6482 (3)	0.1010 (3)	0.9093 (2)	0.0500 (6)	
C7	0.6383 (3)	0.2053 (3)	1.0069 (2)	0.0585 (7)	
H7A	0.7270	0.2394	1.0713	0.070*	
C8	0.5036 (3)	0.2610 (2)	1.0133 (2)	0.0507 (6)	
C9	0.4962 (3)	0.3661 (3)	1.1128 (2)	0.0661 (7)	
H9A	0.5863	0.4026	1.1760	0.079*	
C10	0.3595 (4)	0.4164 (3)	1.1192 (2)	0.0740 (8)	
H10A	0.3573	0.4873	1.1856	0.089*	
C11	0.2231 (4)	0.3603 (3)	1.0251 (2)	0.0670 (7)	
H11A	0.1291	0.3927	1.0305	0.080*	
C12	0.2252 (3)	0.2595 (3)	0.9262 (2)	0.0542 (6)	
H12A	0.1330	0.2245	0.8647	0.065*	
C13	0.3654 (3)	0.2069 (2)	0.91520 (19)	0.0456 (6)	
C14	0.3744 (3)	0.1041 (2)	0.81509 (19)	0.0450 (6)	
C15	0.2306 (3)	0.0455 (3)	0.7113 (2)	0.0511 (6)	
C16	0.1929 (3)	0.1394 (3)	0.6318 (2)	0.0578 (7)	
H16A	0.0974	0.1018	0.5734	0.069*	
C17	0.2810 (3)	0.2731 (3)	0.6344 (2)	0.0512 (6)	
H17A	0.3750	0.3112	0.6943	0.061*	
C18	0.2472 (3)	0.3663 (3)	0.5544 (2)	0.0496 (6)	
C19	0.3340 (3)	0.5069 (3)	0.5521 (2)	0.0552 (7)	
H19A	0.4298	0.5625	0.6057	0.066*	
C20	0.2550 (3)	0.5523 (3)	0.4566 (2)	0.0518 (6)	
C21	0.2817 (3)	0.6796 (3)	0.4082 (2)	0.0659 (8)	
H21A	0.3726	0.7595	0.4423	0.079*	
C22	0.1735 (4)	0.6848 (3)	0.3112 (3)	0.0679 (8)	
H22A	0.1904	0.7692	0.2795	0.081*	
C23	0.0375 (4)	0.5655 (3)	0.2584 (2)	0.0733 (8)	
H23A	−0.0341	0.5721	0.1917	0.088*	
C24	0.0059 (3)	0.4386 (3)	0.3016 (2)	0.0592 (7)	
H24A	−0.0848	0.3591	0.2659	0.071*	
C25	0.1152 (3)	0.4350 (3)	0.4005 (2)	0.0500 (6)	

### (E)-1-(Anthracen-9-yl)-3-(3H-indol-2-yl)prop-2-en-1-one (I) Atomic displacement parameters (Å^2^)

**Table:**

	*U*^11^	*U*^22^	*U*^33^	*U*^12^	*U*^13^	*U*^23^
N1	0.0520 (12)	0.0517 (13)	0.0477 (12)	0.0149 (10)	−0.0024 (10)	0.0073 (10)
O1	0.0678 (12)	0.0495 (11)	0.0815 (13)	0.0003 (9)	−0.0138 (10)	0.0080 (9)
C1	0.0471 (14)	0.0451 (13)	0.0460 (14)	0.0126 (11)	0.0020 (11)	0.0126 (11)
C2	0.0585 (17)	0.0599 (16)	0.0684 (18)	0.0203 (13)	0.0026 (14)	0.0043 (14)
C3	0.080 (2)	0.0659 (18)	0.080 (2)	0.0282 (16)	0.0186 (18)	0.0053 (15)
C4	0.0623 (19)	0.0707 (19)	0.099 (2)	0.0304 (15)	0.0174 (18)	0.0289 (18)
C5	0.0483 (16)	0.0729 (18)	0.075 (2)	0.0183 (13)	0.0031 (14)	0.0303 (16)
C6	0.0478 (14)	0.0496 (14)	0.0516 (15)	0.0127 (11)	0.0006 (12)	0.0165 (12)
C7	0.0502 (15)	0.0668 (17)	0.0497 (16)	0.0084 (12)	−0.0084 (12)	0.0147 (13)
C8	0.0534 (15)	0.0484 (14)	0.0424 (14)	0.0075 (11)	−0.0014 (12)	0.0066 (11)
C9	0.0693 (19)	0.0733 (18)	0.0418 (16)	0.0098 (15)	−0.0038 (13)	0.0009 (13)
C10	0.096 (2)	0.0666 (18)	0.0500 (17)	0.0184 (16)	0.0105 (17)	−0.0047 (14)
C11	0.0762 (19)	0.0655 (17)	0.0626 (18)	0.0295 (14)	0.0132 (15)	0.0089 (15)
C12	0.0552 (15)	0.0560 (15)	0.0477 (15)	0.0168 (12)	−0.0032 (12)	0.0098 (12)
C13	0.0511 (14)	0.0417 (13)	0.0409 (13)	0.0114 (11)	0.0009 (11)	0.0092 (11)
C14	0.0472 (14)	0.0405 (13)	0.0410 (14)	0.0094 (10)	−0.0038 (11)	0.0061 (11)
C15	0.0528 (15)	0.0464 (15)	0.0492 (15)	0.0140 (12)	−0.0008 (12)	0.0055 (12)
C16	0.0595 (16)	0.0549 (16)	0.0466 (15)	0.0095 (13)	−0.0127 (12)	0.0057 (12)
C17	0.0494 (14)	0.0595 (16)	0.0372 (13)	0.0127 (12)	−0.0057 (11)	0.0054 (11)
C18	0.0474 (14)	0.0586 (16)	0.0384 (13)	0.0149 (11)	−0.0006 (11)	0.0055 (12)
C19	0.0507 (15)	0.0568 (16)	0.0472 (15)	0.0039 (12)	0.0043 (12)	0.0014 (12)
C20	0.0602 (15)	0.0535 (15)	0.0409 (14)	0.0165 (12)	0.0084 (12)	0.0081 (12)
C21	0.0749 (19)	0.0565 (17)	0.0635 (18)	0.0122 (13)	0.0173 (15)	0.0099 (14)
C22	0.089 (2)	0.0629 (18)	0.0635 (18)	0.0300 (16)	0.0211 (17)	0.0262 (15)
C23	0.083 (2)	0.090 (2)	0.0588 (18)	0.0460 (18)	0.0058 (16)	0.0216 (17)
C24	0.0608 (16)	0.0623 (17)	0.0525 (16)	0.0232 (13)	−0.0049 (13)	0.0113 (13)
C25	0.0566 (15)	0.0505 (14)	0.0469 (14)	0.0231 (12)	0.0088 (12)	0.0104 (12)

### (E)-1-(Anthracen-9-yl)-3-(3H-indol-2-yl)prop-2-en-1-one (I) Geometric parameters (Å, º)

**Table:**

N1—C25	1.370 (3)	C11—C12	1.356 (3)
N1—C18	1.387 (3)	C11—H11A	0.9300
N1—H1A	0.99 (3)	C12—C13	1.414 (3)
O1—C15	1.229 (3)	C12—H12A	0.9300
C1—C14	1.403 (3)	C13—C14	1.395 (3)
C1—C2	1.405 (3)	C14—C15	1.504 (3)
C1—C6	1.425 (3)	C15—C16	1.441 (3)
C2—C3	1.353 (3)	C16—C17	1.311 (3)
C2—H2A	0.9300	C16—H16A	0.9300
C3—C4	1.395 (4)	C17—C18	1.431 (3)
C3—H3A	0.9300	C17—H17A	0.9300
C4—C5	1.357 (4)	C18—C19	1.377 (3)
C4—H4A	0.9300	C19—C20	1.392 (3)
C5—C6	1.409 (3)	C19—H19A	0.9300
C5—H5A	0.9300	C20—C21	1.406 (3)
C6—C7	1.386 (3)	C20—C25	1.410 (3)
C7—C8	1.380 (3)	C21—C22	1.357 (4)
C7—H7A	0.9300	C21—H21A	0.9300
C8—C9	1.398 (3)	C22—C23	1.394 (4)
C8—C13	1.433 (3)	C22—H22A	0.9300
C9—C10	1.366 (4)	C23—C24	1.370 (4)
C9—H9A	0.9300	C23—H23A	0.9300
C10—C11	1.397 (4)	C24—C25	1.374 (3)
C10—H10A	0.9300	C24—H24A	0.9300
			
C25—N1—C18	108.5 (2)	C14—C13—C12	123.3 (2)
C25—N1—H1A	126.2 (18)	C14—C13—C8	118.9 (2)
C18—N1—H1A	125.3 (18)	C12—C13—C8	117.7 (2)
C14—C1—C2	123.0 (2)	C13—C14—C1	121.6 (2)
C14—C1—C6	119.2 (2)	C13—C14—C15	119.8 (2)
C2—C1—C6	117.8 (2)	C1—C14—C15	118.6 (2)
C3—C2—C1	121.2 (3)	O1—C15—C16	120.5 (2)
C3—C2—H2A	119.4	O1—C15—C14	119.5 (2)
C1—C2—H2A	119.4	C16—C15—C14	120.0 (2)
C2—C3—C4	121.1 (3)	C17—C16—C15	125.9 (2)
C2—C3—H3A	119.5	C17—C16—H16A	117.0
C4—C3—H3A	119.5	C15—C16—H16A	117.0
C5—C4—C3	119.8 (3)	C16—C17—C18	126.6 (2)
C5—C4—H4A	120.1	C16—C17—H17A	116.7
C3—C4—H4A	120.1	C18—C17—H17A	116.7
C4—C5—C6	120.9 (3)	C19—C18—N1	107.9 (2)
C4—C5—H5A	119.5	C19—C18—C17	129.5 (2)
C6—C5—H5A	119.5	N1—C18—C17	122.6 (2)
C7—C6—C5	122.4 (2)	C18—C19—C20	108.9 (2)
C7—C6—C1	118.4 (2)	C18—C19—H19A	125.5
C5—C6—C1	119.2 (2)	C20—C19—H19A	125.5
C8—C7—C6	123.3 (2)	C19—C20—C21	136.1 (2)
C8—C7—H7A	118.3	C19—C20—C25	106.4 (2)
C6—C7—H7A	118.3	C21—C20—C25	117.5 (2)
C7—C8—C9	122.3 (2)	C22—C21—C20	119.4 (2)
C7—C8—C13	118.6 (2)	C22—C21—H21A	120.3
C9—C8—C13	119.1 (2)	C20—C21—H21A	120.3
C10—C9—C8	121.5 (2)	C21—C22—C23	121.0 (3)
C10—C9—H9A	119.3	C21—C22—H22A	119.5
C8—C9—H9A	119.3	C23—C22—H22A	119.5
C9—C10—C11	119.4 (2)	C24—C23—C22	122.1 (3)
C9—C10—H10A	120.3	C24—C23—H23A	119.0
C11—C10—H10A	120.3	C22—C23—H23A	119.0
C12—C11—C10	121.2 (3)	C23—C24—C25	116.5 (2)
C12—C11—H11A	119.4	C23—C24—H24A	121.8
C10—C11—H11A	119.4	C25—C24—H24A	121.8
C11—C12—C13	121.1 (2)	N1—C25—C24	128.1 (2)
C11—C12—H12A	119.5	N1—C25—C20	108.3 (2)
C13—C12—H12A	119.5	C24—C25—C20	123.6 (2)
			
C14—C1—C2—C3	179.9 (2)	C6—C1—C14—C13	−0.6 (3)
C6—C1—C2—C3	0.2 (4)	C2—C1—C14—C15	2.0 (3)
C1—C2—C3—C4	0.9 (4)	C6—C1—C14—C15	−178.34 (19)
C2—C3—C4—C5	−1.4 (4)	C13—C14—C15—O1	−108.1 (3)
C3—C4—C5—C6	0.6 (4)	C1—C14—C15—O1	69.6 (3)
C4—C5—C6—C7	−179.4 (2)	C13—C14—C15—C16	72.7 (3)
C4—C5—C6—C1	0.5 (4)	C1—C14—C15—C16	−109.5 (2)
C14—C1—C6—C7	−0.7 (3)	O1—C15—C16—C17	−173.4 (2)
C2—C1—C6—C7	179.0 (2)	C14—C15—C16—C17	5.7 (4)
C14—C1—C6—C5	179.36 (19)	C15—C16—C17—C18	178.5 (2)
C2—C1—C6—C5	−1.0 (3)	C25—N1—C18—C19	0.3 (3)
C5—C6—C7—C8	−179.6 (2)	C25—N1—C18—C17	−179.3 (2)
C1—C6—C7—C8	0.4 (4)	C16—C17—C18—C19	−180.0 (2)
C6—C7—C8—C9	179.9 (2)	C16—C17—C18—N1	−0.5 (4)
C6—C7—C8—C13	1.1 (4)	N1—C18—C19—C20	−0.5 (3)
C7—C8—C9—C10	−177.8 (2)	C17—C18—C19—C20	179.0 (2)
C13—C8—C9—C10	1.0 (4)	C18—C19—C20—C21	−179.9 (3)
C8—C9—C10—C11	0.9 (4)	C18—C19—C20—C25	0.6 (3)
C9—C10—C11—C12	−1.6 (4)	C19—C20—C21—C22	−179.4 (3)
C10—C11—C12—C13	0.4 (4)	C25—C20—C21—C22	0.1 (3)
C11—C12—C13—C14	−179.6 (2)	C20—C21—C22—C23	−0.6 (4)
C11—C12—C13—C8	1.4 (3)	C21—C22—C23—C24	0.4 (4)
C7—C8—C13—C14	−2.3 (3)	C22—C23—C24—C25	0.3 (4)
C9—C8—C13—C14	178.8 (2)	C18—N1—C25—C24	179.5 (2)
C7—C8—C13—C12	176.7 (2)	C18—N1—C25—C20	0.1 (2)
C9—C8—C13—C12	−2.1 (3)	C23—C24—C25—N1	180.0 (2)
C12—C13—C14—C1	−176.9 (2)	C23—C24—C25—C20	−0.7 (4)
C8—C13—C14—C1	2.1 (3)	C19—C20—C25—N1	−0.4 (3)
C12—C13—C14—C15	0.8 (3)	C21—C20—C25—N1	180.0 (2)
C8—C13—C14—C15	179.82 (19)	C19—C20—C25—C24	−179.8 (2)
C2—C1—C14—C13	179.7 (2)	C21—C20—C25—C24	0.5 (3)

### (E)-1-(Anthracen-9-yl)-3-(3H-indol-2-yl)prop-2-en-1-one (I) Hydrogen-bond geometry (Å, º)

Cg1 and Cg2 are the centroids of the C20–C25 and C1–C6 rings, respectively.

**Table:**

*D*—H···*A*	*D*—H	H···*A*	*D*···*A*	*D*—H···*A*
N1—H1*A*···O1^i^	0.99 (3)	2.03 (3)	2.885 (3)	143 (3)
C10—H10*A*···*Cg*1^ii^	0.93	2.91	3.735 (3)	142
C22—H22*A*···*Cg*2^iii^	0.93	2.75	3.643 (3)	160

Symmetry codes: (i) −*x*, −*y*, −*z*+1; (ii) *x*, *y*, *z*+1; (iii) −*x*+1, −*y*+1, −*z*+1.

### \ (E)-1-(Anthracen-9-yl)-3-[4-(dimethylamino)naphthalen-1-yl]prop-\ 2-en-1-one (II) Crystal data

**Table:**

C_29_H_23_NO	*F*(000) = 848
*M**_r_* = 401.48	*D*_x_ = 1.225 Mg m^−^^3^
Monoclinic, *P*2_1_/*c*	Mo *K*α radiation, λ = 0.71073 Å
*a* = 12.6997 (7) Å	Cell parameters from 9967 reflections
*b* = 11.8029 (7) Å	θ = 2.2–29.9°
*c* = 18.3997 (9) Å	µ = 0.07 mm^−^^1^
β = 127.901 (3)°	*T* = 296 K
*V* = 2176.3 (2) Å^3^	Plate, brown
*Z* = 4	0.62 × 0.54 × 0.23 mm

### \ (E)-1-(Anthracen-9-yl)-3-[4-(dimethylamino)naphthalen-1-yl]prop-\ 2-en-1-one (II) Data collection

**Table:**

Bruker SMART APEXII Duo CCD diffractometer	3215 reflections with *I* > 2σ(*I*)
Radiation source: fine-focus sealed tube	*R*_int_ = 0.073
φ and ω scans	θ_max_ = 30.1°, θ_min_ = 2.0°
Absorption correction: multi-scan (*SADABS*; Bruker, 2009)	*h* = −17→17
*T*_min_ = 0.655, *T*_max_ = 0.946	*k* = −16→16
81111 measured reflections	*l* = −25→25
6334 independent reflections	

### \ (E)-1-(Anthracen-9-yl)-3-[4-(dimethylamino)naphthalen-1-yl]prop-\ 2-en-1-one (II) Refinement

**Table:**

Refinement on *F*^2^	Primary atom site location: structure-invariant direct methods
Least-squares matrix: full	Hydrogen site location: inferred from neighbouring sites
*R*[*F*^2^ > 2σ(*F*^2^)] = 0.075	H-atom parameters constrained
*wR*(*F*^2^) = 0.222	*w* = 1/[σ^2^(*F*_o_^2^) + (0.0755*P*)^2^ + 1.058*P*] where *P* = (*F*_o_^2^ + 2*F*_c_^2^)/3
*S* = 0.97	(Δ/σ)_max_ < 0.001
6334 reflections	Δρ_max_ = 0.34 e Å^−^^3^
282 parameters	Δρ_min_ = −0.22 e Å^−^^3^
0 restraints	

### \ (E)-1-(Anthracen-9-yl)-3-[4-(dimethylamino)naphthalen-1-yl]prop-\ 2-en-1-one (II) Special details

**Table:**

Experimental. The following wavelength and cell were deduced by SADABS from the direction cosines etc. They are given here for emergency use only: CELL 0.71076 11.771 12.680 14.543 95.498 90.095 89.819
Geometry. All esds (except the esd in the dihedral angle between two l.s. planes) are estimated using the full covariance matrix. The cell esds are taken into account individually in the estimation of esds in distances, angles and torsion angles; correlations between esds in cell parameters are only used when they are defined by crystal symmetry. An approximate (isotropic) treatment of cell esds is used for estimating esds involving l.s. planes.

### \ (E)-1-(Anthracen-9-yl)-3-[4-(dimethylamino)naphthalen-1-yl]prop-\ 2-en-1-one (II) Fractional atomic coordinates and isotropic or equivalent isotropic displacement parameters (Å^2^)

**Table:**

	*x*	*y*	*z*	*U*_iso_*/*U*_eq_	
O1	0.5794 (2)	0.2280 (3)	0.80943 (14)	0.1541 (13)	
N1	−0.0318 (3)	0.71428 (17)	0.69994 (17)	0.0861 (7)	
C1	0.4513 (2)	0.2362 (3)	0.59917 (16)	0.0774 (8)	
C2	0.5320 (3)	0.3315 (3)	0.6204 (2)	0.0970 (9)	
H2A	0.5618	0.3761	0.6714	0.116*	
C3	0.5668 (4)	0.3584 (4)	0.5640 (3)	0.1309 (15)	
H3A	0.6190	0.4218	0.5764	0.157*	
C4	0.5215 (6)	0.2874 (6)	0.4864 (4)	0.159 (2)	
H4A	0.5458	0.3050	0.4492	0.191*	
C5	0.4470 (5)	0.1985 (6)	0.4668 (3)	0.153 (2)	
H5A	0.4210	0.1538	0.4167	0.184*	
C6	0.4051 (4)	0.1687 (4)	0.5191 (2)	0.1047 (11)	
C7	0.3230 (4)	0.0754 (4)	0.4982 (3)	0.1251 (14)	
H7A	0.2948	0.0317	0.4471	0.150*	
C8	0.2820 (4)	0.0451 (3)	0.5488 (3)	0.1166 (12)	
C9	0.1921 (5)	−0.0521 (4)	0.5233 (4)	0.1556 (18)	
H9A	0.1595	−0.0960	0.4715	0.187*	
C10	0.1600 (7)	−0.0736 (4)	0.5786 (6)	0.190 (3)	
H10A	0.1036	−0.1346	0.5635	0.228*	
C11	0.2035 (6)	−0.0129 (4)	0.6559 (5)	0.177 (2)	
H11A	0.1758	−0.0322	0.6907	0.212*	
C12	0.2862 (4)	0.0742 (3)	0.6806 (3)	0.1244 (13)	
H12A	0.3174	0.1138	0.7341	0.149*	
C13	0.3288 (3)	0.1091 (3)	0.6275 (2)	0.0890 (8)	
C14	0.4125 (2)	0.2016 (2)	0.65222 (16)	0.0701 (6)	
C15	0.4711 (2)	0.2598 (3)	0.74177 (17)	0.0832 (8)	
C16	0.4021 (2)	0.3514 (2)	0.74860 (15)	0.0717 (7)	
H16A	0.4497	0.3957	0.8016	0.086*	
C17	0.27397 (19)	0.37585 (18)	0.68314 (14)	0.0551 (5)	
H17A	0.2302	0.3333	0.6294	0.066*	
C18	0.19498 (18)	0.46181 (16)	0.68650 (13)	0.0509 (4)	
C19	0.2555 (2)	0.5478 (2)	0.75005 (17)	0.0700 (6)	
H19A	0.3482	0.5507	0.7909	0.084*	
C20	0.1823 (3)	0.6310 (2)	0.75520 (19)	0.0786 (7)	
H20A	0.2273	0.6872	0.7998	0.094*	
C21	0.0457 (2)	0.63188 (17)	0.69611 (17)	0.0637 (6)	
C22	−0.0224 (2)	0.54867 (16)	0.62504 (14)	0.0519 (5)	
C23	−0.1629 (2)	0.5519 (2)	0.55500 (16)	0.0655 (6)	
H23A	−0.2122	0.6104	0.5548	0.079*	
C24	−0.2264 (2)	0.4717 (2)	0.48864 (16)	0.0726 (6)	
H24A	−0.3183	0.4762	0.4431	0.087*	
C25	−0.1553 (2)	0.3828 (2)	0.48809 (15)	0.0674 (6)	
H25A	−0.2002	0.3263	0.4437	0.081*	
C26	−0.01990 (19)	0.37794 (17)	0.55242 (13)	0.0545 (5)	
H26A	0.0263	0.3183	0.5507	0.065*	
C27	0.05179 (18)	0.46122 (15)	0.62158 (12)	0.0463 (4)	
C28	0.0349 (5)	0.8198 (3)	0.7467 (3)	0.1426 (17)	
H28A	−0.0297	0.8738	0.7360	0.214*	
H28B	0.0795	0.8491	0.7234	0.214*	
H28C	0.0992	0.8061	0.8117	0.214*	
C29	−0.1074 (3)	0.6725 (3)	0.7296 (2)	0.0987 (9)	
H29A	−0.1747	0.7268	0.7140	0.148*	
H29B	−0.0484	0.6612	0.7951	0.148*	
H29C	−0.1491	0.6019	0.6994	0.148*	

### \ (E)-1-(Anthracen-9-yl)-3-[4-(dimethylamino)naphthalen-1-yl]prop-\ 2-en-1-one (II) Atomic displacement parameters (Å^2^)

**Table:**

	*U*^11^	*U*^22^	*U*^33^	*U*^12^	*U*^13^	*U*^23^
O1	0.0952 (15)	0.257 (3)	0.0676 (12)	0.0978 (19)	0.0283 (12)	0.0303 (16)
N1	0.1280 (18)	0.0587 (12)	0.1233 (18)	0.0009 (12)	0.1033 (17)	−0.0095 (12)
C1	0.0679 (14)	0.107 (2)	0.0650 (14)	0.0458 (14)	0.0448 (12)	0.0316 (14)
C2	0.0718 (16)	0.124 (3)	0.100 (2)	0.0296 (17)	0.0552 (16)	0.0380 (19)
C3	0.102 (2)	0.168 (4)	0.144 (3)	0.037 (2)	0.087 (3)	0.067 (3)
C4	0.172 (5)	0.236 (7)	0.128 (4)	0.054 (4)	0.121 (4)	0.061 (4)
C5	0.173 (5)	0.224 (6)	0.106 (3)	0.065 (4)	0.108 (3)	0.035 (4)
C6	0.110 (2)	0.141 (3)	0.0816 (19)	0.057 (2)	0.0677 (19)	0.027 (2)
C7	0.128 (3)	0.133 (3)	0.096 (2)	0.044 (3)	0.060 (2)	−0.014 (2)
C8	0.124 (3)	0.097 (3)	0.139 (3)	0.035 (2)	0.086 (3)	−0.002 (2)
C9	0.166 (4)	0.105 (3)	0.202 (5)	0.011 (3)	0.116 (4)	−0.036 (3)
C10	0.231 (6)	0.102 (3)	0.322 (9)	0.001 (4)	0.212 (7)	−0.029 (5)
C11	0.249 (6)	0.101 (3)	0.295 (7)	0.013 (4)	0.225 (6)	0.017 (4)
C12	0.154 (3)	0.090 (2)	0.187 (4)	0.023 (2)	0.133 (3)	0.030 (2)
C13	0.0925 (19)	0.0826 (19)	0.103 (2)	0.0389 (16)	0.0656 (17)	0.0218 (16)
C14	0.0639 (13)	0.0826 (16)	0.0683 (14)	0.0342 (12)	0.0428 (12)	0.0220 (12)
C15	0.0605 (13)	0.130 (2)	0.0573 (13)	0.0352 (14)	0.0351 (12)	0.0242 (14)
C16	0.0559 (12)	0.1080 (19)	0.0481 (11)	0.0097 (12)	0.0304 (10)	0.0009 (12)
C17	0.0516 (10)	0.0662 (13)	0.0515 (10)	−0.0004 (9)	0.0338 (9)	0.0006 (9)
C18	0.0519 (10)	0.0542 (11)	0.0537 (10)	−0.0068 (8)	0.0360 (9)	−0.0040 (9)
C19	0.0597 (12)	0.0789 (15)	0.0775 (14)	−0.0222 (11)	0.0453 (12)	−0.0239 (12)
C20	0.0923 (18)	0.0701 (15)	0.0977 (18)	−0.0313 (13)	0.0706 (16)	−0.0365 (13)
C21	0.0864 (15)	0.0485 (11)	0.0868 (15)	−0.0081 (10)	0.0686 (14)	−0.0084 (11)
C22	0.0627 (11)	0.0454 (10)	0.0638 (11)	0.0018 (9)	0.0471 (10)	0.0066 (9)
C23	0.0664 (13)	0.0663 (14)	0.0737 (14)	0.0190 (11)	0.0480 (12)	0.0162 (12)
C24	0.0526 (12)	0.0906 (17)	0.0614 (13)	0.0106 (12)	0.0283 (11)	0.0114 (13)
C25	0.0574 (12)	0.0724 (15)	0.0569 (12)	−0.0064 (11)	0.0273 (10)	−0.0045 (10)
C26	0.0534 (11)	0.0522 (11)	0.0524 (11)	0.0007 (9)	0.0297 (9)	−0.0019 (9)
C27	0.0507 (10)	0.0427 (10)	0.0513 (10)	−0.0028 (8)	0.0343 (9)	0.0018 (8)
C28	0.216 (4)	0.072 (2)	0.238 (5)	−0.027 (2)	0.189 (4)	−0.049 (3)
C29	0.114 (2)	0.106 (2)	0.122 (2)	0.0084 (18)	0.096 (2)	−0.0032 (19)

### \ (E)-1-(Anthracen-9-yl)-3-[4-(dimethylamino)naphthalen-1-yl]prop-\ 2-en-1-one (II) Geometric parameters (Å, º)

**Table:**

O1—C15	1.215 (3)	C14—C15	1.494 (4)
N1—C21	1.416 (3)	C15—C16	1.446 (3)
N1—C29	1.452 (3)	C16—C17	1.331 (3)
N1—C28	1.454 (4)	C16—H16A	0.9300
C1—C14	1.398 (3)	C17—C18	1.454 (3)
C1—C2	1.407 (4)	C17—H17A	0.9300
C1—C6	1.445 (4)	C18—C19	1.373 (3)
C2—C3	1.391 (5)	C18—C27	1.437 (3)
C2—H2A	0.9300	C19—C20	1.395 (3)
C3—C4	1.436 (7)	C19—H19A	0.9300
C3—H3A	0.9300	C20—C21	1.369 (3)
C4—C5	1.306 (7)	C20—H20A	0.9300
C4—H4A	0.9300	C21—C22	1.426 (3)
C5—C6	1.399 (5)	C22—C23	1.422 (3)
C5—H5A	0.9300	C22—C27	1.425 (3)
C6—C7	1.399 (5)	C23—C24	1.352 (3)
C7—C8	1.363 (5)	C23—H23A	0.9300
C7—H7A	0.9300	C24—C25	1.389 (3)
C8—C13	1.403 (5)	C24—H24A	0.9300
C8—C9	1.478 (6)	C25—C26	1.364 (3)
C9—C10	1.331 (7)	C25—H25A	0.9300
C9—H9A	0.9300	C26—C27	1.411 (3)
C10—C11	1.369 (8)	C26—H26A	0.9300
C10—H10A	0.9300	C28—H28A	0.9600
C11—C12	1.334 (6)	C28—H28B	0.9600
C11—H11A	0.9300	C28—H28C	0.9600
C12—C13	1.437 (4)	C29—H29A	0.9600
C12—H12A	0.9300	C29—H29B	0.9600
C13—C14	1.390 (4)	C29—H29C	0.9600
			
C21—N1—C29	115.2 (2)	C17—C16—C15	123.6 (2)
C21—N1—C28	116.6 (2)	C17—C16—H16A	118.2
C29—N1—C28	110.3 (2)	C15—C16—H16A	118.2
C14—C1—C2	123.4 (3)	C16—C17—C18	127.2 (2)
C14—C1—C6	117.1 (3)	C16—C17—H17A	116.4
C2—C1—C6	119.6 (3)	C18—C17—H17A	116.4
C3—C2—C1	119.1 (4)	C19—C18—C27	118.18 (18)
C3—C2—H2A	120.4	C19—C18—C17	120.80 (18)
C1—C2—H2A	120.4	C27—C18—C17	120.99 (17)
C2—C3—C4	119.5 (4)	C18—C19—C20	122.0 (2)
C2—C3—H3A	120.3	C18—C19—H19A	119.0
C4—C3—H3A	120.3	C20—C19—H19A	119.0
C5—C4—C3	121.5 (4)	C21—C20—C19	121.6 (2)
C5—C4—H4A	119.3	C21—C20—H20A	119.2
C3—C4—H4A	119.3	C19—C20—H20A	119.2
C4—C5—C6	122.0 (5)	C20—C21—N1	123.1 (2)
C4—C5—H5A	119.0	C20—C21—C22	118.84 (19)
C6—C5—H5A	119.0	N1—C21—C22	118.0 (2)
C5—C6—C7	122.9 (4)	C23—C22—C27	118.38 (19)
C5—C6—C1	118.4 (4)	C23—C22—C21	121.96 (19)
C7—C6—C1	118.7 (3)	C27—C22—C21	119.63 (18)
C8—C7—C6	123.6 (4)	C24—C23—C22	121.3 (2)
C8—C7—H7A	118.2	C24—C23—H23A	119.3
C6—C7—H7A	118.2	C22—C23—H23A	119.3
C7—C8—C13	117.8 (4)	C23—C24—C25	120.4 (2)
C7—C8—C9	122.2 (5)	C23—C24—H24A	119.8
C13—C8—C9	120.0 (4)	C25—C24—H24A	119.8
C10—C9—C8	116.5 (5)	C26—C25—C24	120.3 (2)
C10—C9—H9A	121.8	C26—C25—H25A	119.9
C8—C9—H9A	121.8	C24—C25—H25A	119.9
C9—C10—C11	125.2 (6)	C25—C26—C27	121.62 (19)
C9—C10—H10A	117.4	C25—C26—H26A	119.2
C11—C10—H10A	117.4	C27—C26—H26A	119.2
C12—C11—C10	119.2 (5)	C26—C27—C22	117.82 (17)
C12—C11—H11A	120.4	C26—C27—C18	122.62 (17)
C10—C11—H11A	120.4	C22—C27—C18	119.55 (17)
C11—C12—C13	122.4 (5)	N1—C28—H28A	109.5
C11—C12—H12A	118.8	N1—C28—H28B	109.5
C13—C12—H12A	118.8	H28A—C28—H28B	109.5
C14—C13—C8	121.0 (3)	N1—C28—H28C	109.5
C14—C13—C12	122.3 (3)	H28A—C28—H28C	109.5
C8—C13—C12	116.8 (4)	H28B—C28—H28C	109.5
C13—C14—C1	121.8 (3)	N1—C29—H29A	109.5
C13—C14—C15	119.0 (2)	N1—C29—H29B	109.5
C1—C14—C15	119.0 (3)	H29A—C29—H29B	109.5
O1—C15—C16	120.4 (3)	N1—C29—H29C	109.5
O1—C15—C14	118.2 (2)	H29A—C29—H29C	109.5
C16—C15—C14	121.42 (19)	H29B—C29—H29C	109.5
			
C14—C1—C2—C3	179.2 (2)	C13—C14—C15—C16	−88.5 (3)
C6—C1—C2—C3	−0.6 (4)	C1—C14—C15—C16	96.4 (3)
C1—C2—C3—C4	−1.0 (5)	O1—C15—C16—C17	−166.2 (3)
C2—C3—C4—C5	0.7 (7)	C14—C15—C16—C17	13.8 (4)
C3—C4—C5—C6	1.2 (8)	C15—C16—C17—C18	176.2 (2)
C4—C5—C6—C7	178.6 (5)	C16—C17—C18—C19	17.4 (3)
C4—C5—C6—C1	−2.7 (7)	C16—C17—C18—C27	−164.6 (2)
C14—C1—C6—C5	−177.4 (3)	C27—C18—C19—C20	2.4 (3)
C2—C1—C6—C5	2.4 (4)	C17—C18—C19—C20	−179.6 (2)
C14—C1—C6—C7	1.4 (4)	C18—C19—C20—C21	−0.8 (4)
C2—C1—C6—C7	−178.9 (3)	C19—C20—C21—N1	179.4 (2)
C5—C6—C7—C8	179.6 (4)	C19—C20—C21—C22	−3.0 (4)
C1—C6—C7—C8	1.0 (5)	C29—N1—C21—C20	−111.2 (3)
C6—C7—C8—C13	−2.1 (5)	C28—N1—C21—C20	20.5 (4)
C6—C7—C8—C9	178.2 (4)	C29—N1—C21—C22	71.1 (3)
C7—C8—C9—C10	179.6 (5)	C28—N1—C21—C22	−157.1 (3)
C13—C8—C9—C10	−0.1 (7)	C20—C21—C22—C23	−172.8 (2)
C8—C9—C10—C11	0.1 (9)	N1—C21—C22—C23	5.0 (3)
C9—C10—C11—C12	−1.0 (10)	C20—C21—C22—C27	5.2 (3)
C10—C11—C12—C13	1.8 (7)	N1—C21—C22—C27	−177.07 (18)
C7—C8—C13—C14	1.0 (4)	C27—C22—C23—C24	2.8 (3)
C9—C8—C13—C14	−179.4 (3)	C21—C22—C23—C24	−179.2 (2)
C7—C8—C13—C12	−178.9 (3)	C22—C23—C24—C25	0.8 (3)
C9—C8—C13—C12	0.8 (5)	C23—C24—C25—C26	−2.6 (4)
C11—C12—C13—C14	178.5 (4)	C24—C25—C26—C27	0.7 (3)
C11—C12—C13—C8	−1.7 (5)	C25—C26—C27—C22	2.9 (3)
C8—C13—C14—C1	1.4 (4)	C25—C26—C27—C18	−176.00 (19)
C12—C13—C14—C1	−178.8 (2)	C23—C22—C27—C26	−4.5 (3)
C8—C13—C14—C15	−173.7 (2)	C21—C22—C27—C26	177.48 (17)
C12—C13—C14—C15	6.2 (4)	C23—C22—C27—C18	174.38 (17)
C2—C1—C14—C13	177.8 (2)	C21—C22—C27—C18	−3.6 (3)
C6—C1—C14—C13	−2.5 (3)	C19—C18—C27—C26	178.72 (19)
C2—C1—C14—C15	−7.2 (3)	C17—C18—C27—C26	0.7 (3)
C6—C1—C14—C15	172.5 (2)	C19—C18—C27—C22	−0.1 (3)
C13—C14—C15—O1	91.5 (3)	C17—C18—C27—C22	−178.11 (17)
C1—C14—C15—O1	−83.6 (4)		

### \ (E)-1-(Anthracen-9-yl)-3-[4-(dimethylamino)naphthalen-1-yl]prop-\ 2-en-1-one (II) Hydrogen-bond geometry (Å, º)

**Table:**

*D*—H···*A*	*D*—H	H···*A*	*D*···*A*	*D*—H···*A*
C25—H25*A*···O1^i^	0.93	2.42	3.203 (3)	142

Symmetry code: (i) *x*−1, −*y*+1/2, *z*−1/2.

## References

[bb1] Abdullah, A. A., Hassan, N. H. H., Arshad, S., Khalib, N. C. & Razak, I. A. (2016). *Acta Cryst.* E**72**, 648–651. 10.1107/S2056989016005028 PMC4908534 27308010

[bb2] Agrahari, A., Wagers, P. O., Schildcrout, S. M., Masnovi, J. & Youngs, W. J. (2015). *Acta Cryst.* E**71**, 357–359. 10.1107/S2056989015004090 PMC4438842 26029389

[bb24] Bruker (2009). *APEX2*, *SAINT* and *SADABS*. Bruker AXS Inc., Madison, Wisconsin, USA.

[bb3] Cicogna, F., Ingrosso, G., Lodato, F., Marchetti, F. & Zandomeneghi, M. (2004). *Tetrahedron*, **60**, 11959–11968.

[bb5] D’silva, E. D., Podagatlapalli, G. K., Rao, S. V., Rao, D. N. & Dharmaprakash, S. M. (2011). *Cryst. Growth Des.* **11**, 5326–5369.

[bb6] Ebenezar, J. D., Ramalingam, S., Raja, C. R. & Helan, V. (2013). *J. Theo. Comput. Sci.* **1**, 1–13.

[bb23] Frisch, M.J., Trucks, G.W., Schlegel, H.B., Scuseria, G.E., Robb, M.A., Cheeseman, J. R., Scalmani, G., Barone, V., Mennucci, B., Petersson, G.A., Nakatsuji, H., Caricato, M., Li, X., Hratchian, H.P., Izmaylov, A.F., Bloino, J., Zheng, V., Sonnenberg, J. L., Hada, M., Ehara, M., Toyota, K., Fukuda, R., Hasegawa, J., Ishida, M., Nakajima, T., Honda, Y., Kitao, O., Nakai, H., Vreven, T., Montgomery, J. A., Peralta, J. E., Ogliaro, F., Bearpark, M., Heyd, J. J., Brothers, E., Kudin, K. N., Staroverov, V. N., Kobayashi, R., Normand, J., Raghavachari, K., Rendell, A., Burant, J. C., Iyengar, S. C., Tomasi, J., Cossi, M., Rega, N., Millam, J. M., Klene, M., Knox, J. E., Cross, J. B., Bakken, V., Adamo, C., Jaramillo, J., Gomperts, R., Stratmann, R. E., Yazyev, O., Austin, A. J., Cammi, R., Pomelli, C., Ochterski, J. W., Martin, R. L., Morokuma, K., Zakrzewski, V.G., Voth, G. A., Salvador, P., Dannenberg, J. J., Dapprich, S., Daniels, A. D., Farkas, Ö., Foresman, J. B., Ortiz, J. V., Cioslowski, J. & Fox, D. J. (2009). *Gaussian 09*, Revision A.1. Gaussian, Inc., Wallingford CT, USA.

[bb7] Girisha, M., Yathirajan, H. S., Jasinski, J. P. & Glidewell, C. (2016). *Acta Cryst.* E**72**, 1153–1158. 10.1107/S2056989016011592 PMC4971861 27536402

[bb8] Groom, C. R., Bruno, I. J., Lightfoot, M. P. & Ward, S. C. (2016). *Acta Cryst.* B**72**, 171–179. 10.1107/S2052520616003954 PMC4822653 27048719

[bb9] Harlow, R. L., Loghry, R. A., Williams, H. J. & Simonsen, S. H. (1975). *Acta Cryst.* B**31**, 1344–1350.

[bb10] He, S. G., Tan, L. S., Zheng, Q. & Prasad, P. N. (2008). *Chem. Rev.* **108**, 1245–1330. 10.1021/cr050054x 18361528

[bb11] Jung, Y., , Son, K., Oh, Y. E. & Noh, D. (2008). *Polyhedron*, **27**, 861–867.

[bb12] Sathish, S., Shekar, B. C., Kannan, S. C., Sengodan, R., Dinesh, K. P. B. & Ranjithkumar, R. (2015). *Int. J. Polym. Anal. Charact.* **20**, 29–41.

[bb13] Sheldrick, G. M. (2008). *Acta Cryst.* A**64**, 112–122. 10.1107/S0108767307043930 18156677

[bb14] Sheldrick, G. M. (2015). *Acta Cryst.* C**71**, 3–8.

[bb15] Spek, A. L. (2009). *Acta Cryst.* D**65**, 148–155. 10.1107/S090744490804362X PMC2631630 19171970

[bb16] Tejkiran, P. J., Teja, M. S. B., Kumar, P. S. S., Sankar, P., Philip, R., Naveen, S., Lokanath, N. K. & Rao, G. N. (2016). *J. Photochemistry Photobiology A: Chemistry*, **324**, 233–39.

[bb17] Wu, W., Liu, Y. & Zhu, D. (2010). *Chem. Soc. Rev.* **39**, 1489–1502. 10.1039/b813123f 20419204

[bb19] Zainuri, D. A., Razak, I. A. & Arshad, S. (2018*a*). *Acta Cryst.* E**74**, 492–496. 10.1107/S2056989018003791 PMC5946975 29765753

[bb20] Zainuri, D. A., Razak, I. A. & Arshad, S. (2018*b*). *Acta Cryst.* E**74**, 650–655. 10.1107/S2056989018005467 PMC5947480 29850084

[bb21] Zainuri, D. A., Razak, I. A. & Arshad, S. (2018*c*). *Acta Cryst.* E**74**, 780–785. 10.1107/S2056989018006527 PMC6002829 29951229

[bb22] Zainuri, D. A., Razak, I. A. & Arshad, S. (2018*d*). *Acta Cryst.* E**74**, 1087–1092. 10.1107/S205698901800974X PMC6072983 30116568

