# Supplementary material for: Crystal structures, DFT studies and UV–visible absorption spectra of two anthracenyl chalcone derivatives
Source: Acta Crystallogr E Crystallogr Commun. 2018 Sep 28;74(Pt 10):1491–6. doi: 10.1107/S2056989018013087 (PMC6176452; doi:10.1107/S2056989018013087)
Supplement: Supplementary file 6 [file e-74-01491-sup6.pdf]

(*E*)-1-(anthracen-9-yl)-3-(3*H*-indol-2-yl)prop-2-en-1-one and (*E*)-1-(anthracen-9-yl)-3-(4-(dimethylamino)naphthalen-1-yl)prop-2-en-1-one : Crystal Structure, UV-Vis absorption and Molecular Electrostatic Potential analysis

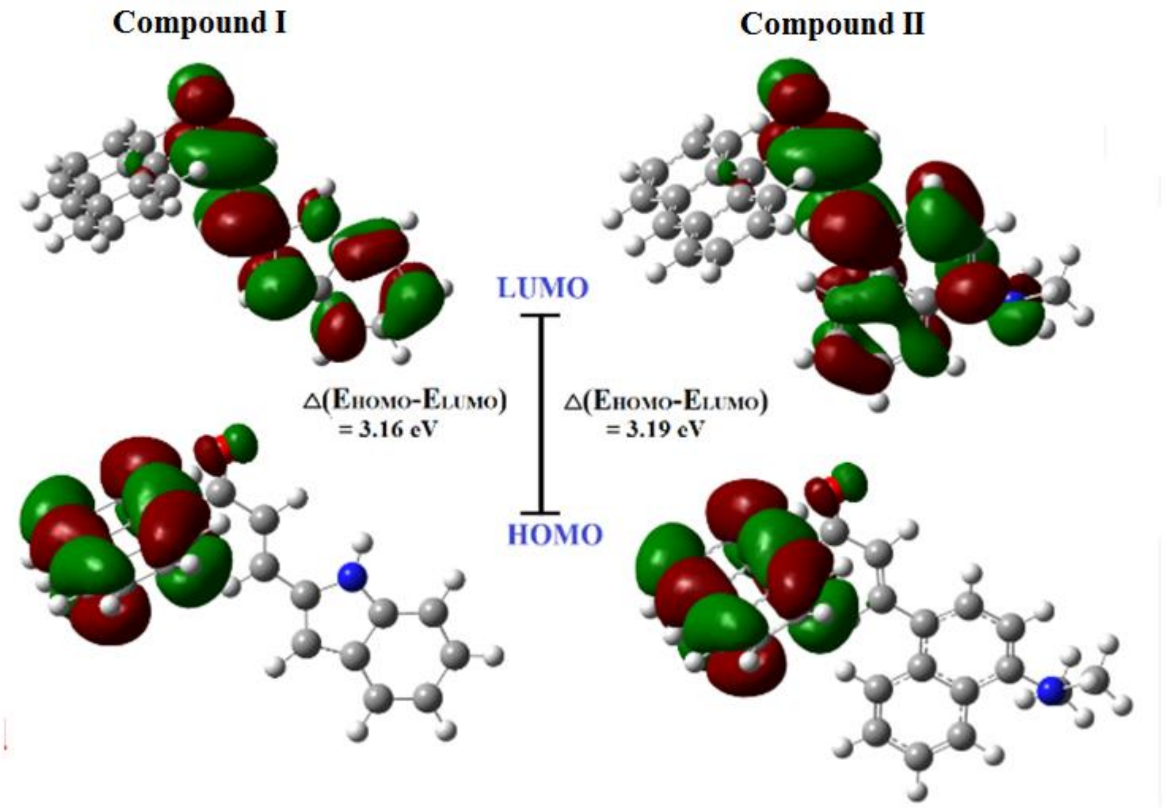

Figure S1 The electron distribution of the HOMO and LUMO energy level **I** and **II.**

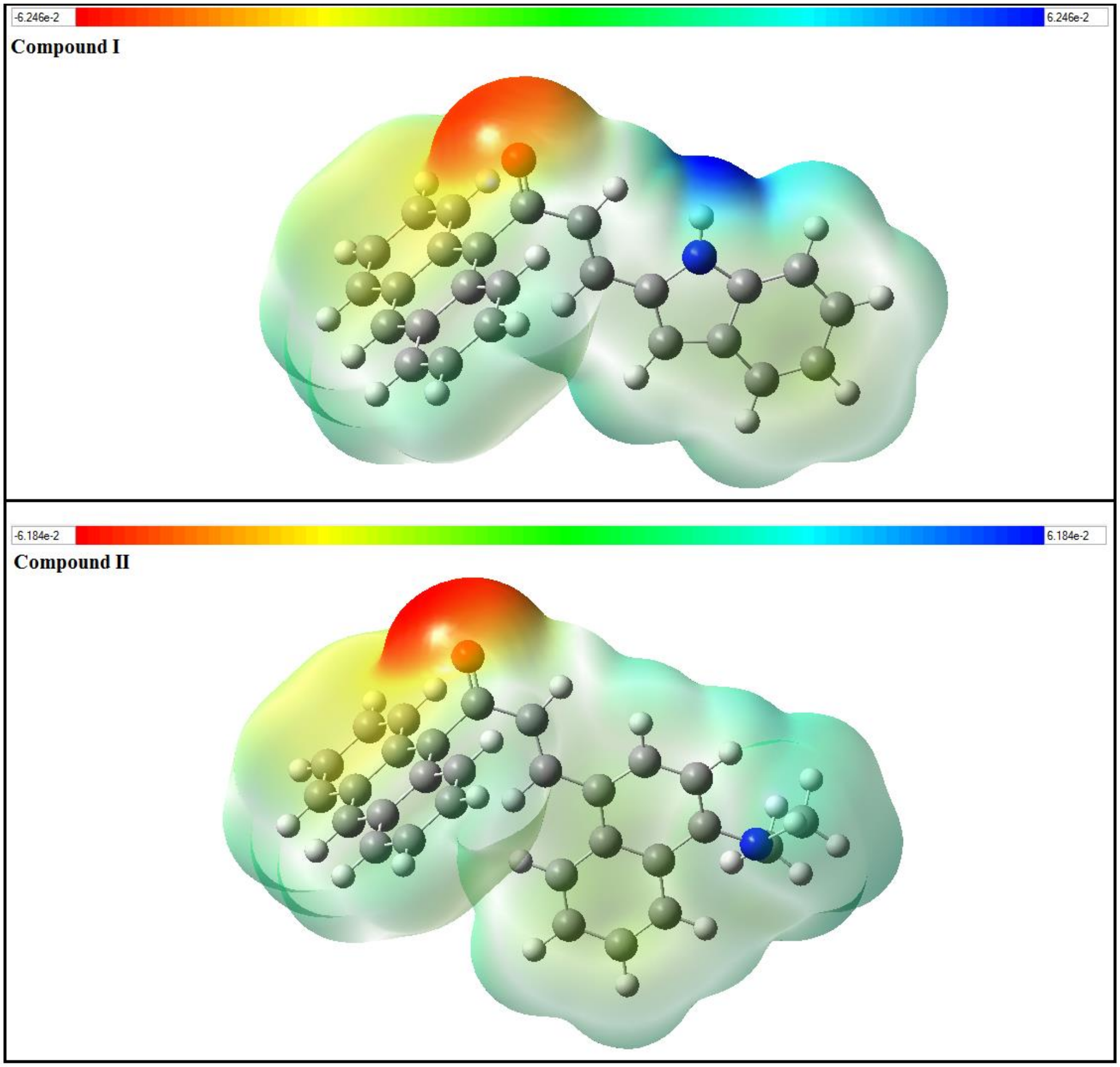

Figure S2 Molecular electrostatic potentials (MEP) mapped on the electron density surface calculated by the DFT/B3LYP method.

Table S1 A Comparison Selected Single Crystal X-Ray data and DFT Geometry Optimized Data for Compounds (**I**) and (**II**).

	**Compound I**		**Compound II**	
**Bonds**	**X-ray (Experimental) (Å^0)**	**DFT (Theoretical) (Å^0)**	**X-ray (Experimental) (Å^0)**	**DFT (Theoretical) (Å^0)**
C15-O1	1.23 (3)	1.22	1.22 (3)	1.22
C1-C14	1.40 (3)	1.41	1.40 (3)	1.41
C1-C2	1.41 (3)	1.43	1.41 (4)	1.43
C2-C3	1.35 (4)	1.37	1.39 (5)	1.37
C3-C4	1.40 (4)	1.42	1.44 (7)	1.42
C4-C5	1.36 (4)	1.37	1.31 (7)	1.37
C5-C6	1.41 (3)	1.43	1.40 (5)	1.43
C6-C7	1.39 (3)	1.40	1.40 (5)	1.40
C7-C8	1.38 (3)	1.40	1.36 (5)	1.40
C8-C9	1.40 (3)	1.43	1.48 (6)	1.43
C9-C10	1.37 (4)	1.37	1.33 (7)	1.37
C10-C11	1.40 (4)	1.42	1.37 (8)	1.42
C11-C12	1.36 (3)	1.37	1.37 (8)	1.37
C12-C13	1.41 (3)	1.43	1.44 (4)	1.43
C13-C14	1.40 (3)	1.41	1.39 (4)	1.41
C14-C15	1.50 (3)	1.52	1.49 (4)	1.52
C15-C16	1.44 (3)	1.47	1.45 (3)	1.47
C16-C17	1.31 (3)	1.35	1.33 (3)	1.35
C17-C18	1.43 (3)	1.43	1.45 (3)	1.46
C14—C15—C16	120.0 (2)	119.21	121.42 (19)	119.42
O1—C15—C14	119.6 (2)	120.15	118.2 (2)	119.82
O1—C15—C16	120.5 (2)	120.64	120.4 (3)	120.76
C15—C16—C17	125.9 (2)	124.41	123.6 (2)	123.98
C16—C17—C18	126.6 (2)	126.80	127.2 (2)	127.08
C1—C14—C15—O1	69.6 (3)	88.85	−83.6 (4)	83.45
C13—C14—C15—O1	72.8 (3)	-88.81	91.5 (3)	-94.45
C1—C14—C15—C16	−109.5 (3)	-91.14	96.4 (3)	-95.95
C13—C14—C15—C16	72.8 (3)	91.19	−88.5 (3)	86.15
O1—C15—C16—C17	−173.3 (2)	180.00	−166.2 (3)	-179.16
C14—C15—C16—C17	5.8 (4)	0.00	13.8 (4)	0.23

C15—C16—C17—C18	178.4 (2)	180.00	176.2 (2)	-178.23
C16—C17—C18—C19	179.9 (2)	-180.00	17.4 (3)	18.67

* B3LPY/6-311++G(d,p)
